# Outcome of patients treated for myelodysplastic syndromes with 5q deletion after failure of lenalidomide therapy

**DOI:** 10.18632/oncotarget.18477

**Published:** 2017-06-14

**Authors:** Thomas Prebet, Thomas Cluzeau, Sophie Park, Mikkael A. Sekeres, Ulrich Germing, Lionel Ades, Uwe Platzbecker, Katharina Gotze, Norbert Vey, Esther Oliva, Mary M. Sugrue, Cecile Bally, Charikleia Kelaidi, Najla Al Ali, Pierre Fenaux, Steven D. Gore, Rami Komrokji

**Affiliations:** ^1^ Moffit Cancer Center, Tampa, Florida, USA; ^2^ Cote d’Azur University, Nice Sophia Antipolis University, Centre Hospitalier Universitaire de Nice, Nice, France; ^3^ U1065, Mediterranean Center of Molecular Medecine, Nice, France; ^4^ Groupe Francophone des Myelodysplasies, Hopital Saint Louis, Paris, France; ^5^ Leukemia Program, Cleveland Clinic, Cleveland, OH, USA; ^6^ Department Hematology, Oncology and Clinical Immunology, Heinrich Heine University, Dusseldorf, Germany; ^7^ Service Hematologie Senior, Hopital Saint Louis, Paris, France; ^8^ Universitäts Klinikum Carl Gustav Carus, Dresden, Germany; ^9^ Klinikum Rechts der Isar, Technische Universität München, München, Germany; ^10^ Departement d’Hematologie, Institut Paoli-Calmettes, Marseille, France; ^11^ Hematology Unit, Azienda Ospedaliera Bianchi Melacrino Morelli, Reggio Calabria, Italy; ^12^ Celgene Corporation, Summit, NJ, USA

**Keywords:** myelodysplasia, outcome, lenalidomide

## Abstract

While lenalidomide (LEN) is the standard of care for the lower-risk myelodysplastic syndromes (MDS) patients with deletion 5q, 35% will not respond to or do not tolerate the drug. Moreover, most of the patients will lose their response after a few years. Defining the outcome of patients with LEN failure and determining the impact of subsequent therapies is therefore important to develop alternative strategies. Based on an international collaboration, we were able to compile a total of 392 patient cases of lower-risk MDS patients with 5q deletion and to analyze their outcome after failure of lenalidomide. The median survival following LEN failure was 23 months. We observed a negative impact on survival of advanced age, higher bone marrow blast count at LEN initiation, progression after LEN failure, and unfavorable cytogenetics. Among the treatment strategies, we observed a relatively prolonged survival of patients treated subsequently with hypomethylating agents and only a limited impact on survival of allogeneic transplantation. In conclusion, our work stresses the relatively short survival of this group of patient and defines the expected baseline for the needed future investigations in this group of patients.

## INTRODUCTION

Myelodysplastic syndromes (MDS) are a heterogeneous group of diseases characterized by ineffective hematopoiesis, peripheral blood cytopenias, and a risk of evolution to acute myeloid leukemia [1]. Over the last decade, hypomethylating agents [2-4] (HMA) and lenalidomide [5, 6] (LEN) changed therapeutic approaches for MDS, offering for the first time treatments able to durably correct cytopenias and potentially to prolong survival. In the case of azacitidine, higher-risk patients experienced prolonged survival when compared to patients treated with conventional care but a vast majority of patients will eventually relapse or experience disease progression [7]. The outcome of patients experiencing failure of HMA is poor and several studies [8, 9] defined the basis on which we are currently trying to build innovative strategies in this setting. Lenalidomide has significant activity in lower-risk MDS harboring deletion (del) 5q. The mechanism of action of the drug involves Cereblon, to which LEN binds [10], and Casein Kinase 1A1 [11]. LEN induces sustained hematological and cytogenetic responses with red blood cells (RBC) transfusion independence in the majority of patients. However, 30 to 40% of patients will not respond to or potentially will not tolerate the drug. Moreover, for responders, the median duration of response ranges is approximately two years [12, 13]. LEN has also been tested in higher-risk disease with del 5q as single agent [14], in combination with HMA [15, 16], or with chemotherapy [17]; however a more limited success was observed. LEN has obtained Food and Drug Administration approval for transfusion-dependent anemia due to low- or intermediate-1-risk MDS associated with a del 5q with or without one additional cytogenetic abnormality, whereas the European Medicine Agency has restricted approval for those with isolated del 5q cytogenetic when other therapeutic options are insufficient or inadequate. The drug is still undergoing additional monitoring due to the lack of sufficient follow-up data.

There is no universally accepted second-line treatment for patients experiencing LEN failure and only a limited number of patients undergo allogeneic transplantation. Several groups of investigators are currently developing strategies for del 5q patients experiencing LEN failure. The use of HMAs may be an option, as well as new drugs such as imetelstat, immunotherapies, or TGF-Beta inhibitors[18, 19] but, to date, there is no standard of care, or ongoing phase III studies. To appropriately design and analyze trials in this population, it would be important to estimate their expected survival with current care options.

This study aimed to answer this question using a large retrospective cohort based on an international multicenter consortium of centers from Europe and North America.

## RESULTS

### Patients’ characteristics

A total of 392 eligible patients treated between 2004 and 2015 and meeting eligibility criteria were included in the present study (see Table 1 for details). A majority of the patients were prospectively enrolled in clinical trials and compassionate use programs (n=261, 66%, MDS-003 and MDS-004 studies, French lenalidomide compassionate use program) while MDS registries patients represented 34% of the total cohort. The number of patients were well balanced between US and Europe (216 and 176 patients respectively). As expected for a population defined by the presence of del 5q, the patients’ characteristics were slightly different from the general population of MDS patients. The median age was only 70y (range [37-95]) and there was a female predominance (2/3 of the patients). At diagnosis, CBC showed a median hemoglobin level of 9.2g/dl (range [3.8-13.6]) but preserved platelet counts (median platelet count: 223G/l, range [17-974]). The majority of patients had low bone marrow blast counts (median: 3%). with only 23% of patients with RAEB-1. Of note, therapy-related MDS represented 16% of the cohort.

**Table 1:** Patients characteristics.

Variable	
**N=**	392
**Median age**	70y (37-95)
**Male Gender**	134 (34%)
**WHO classification**	
RA/ del5q	125 (32%) / 97 (25%)
RARS/RCMD	27 (7%) /41 (10%)
RAEB-1	91 (23%)
Other	11 (3%)
**Therapy related MDS**	61 (16%)
**Median BM blast count (%)**	3 (0-9)
**Deletion (5q)**	
isolated	230 (61%)
Del(5q)+1 aberration	105 (24%)
Complex K including del(5q)	42 (11%)
FISH only	15 (4%)
**RBC TD before LEN**	353 (90%)
**Use of ESA before LEN**	155 (39%)
**IPSS low/intermediate-1**	161 (41%) /231 (59%)
**Use of HMA before LEN**	13 (3%)
**LEN response**	210 (54%)
**LEN initial dose 10mg daily**	296 (76%)
**LEN duration**	9.5m (1-68)

M/F: male/female, RA: refractory anemia, del5q: 5q syndrome, RARS: refractory anemia with ring sideroblasts, RCMD: Refractory cytopenia with multilineage dysplasia, RAEB: Refractory anemia with excess of blast, BM: bone marrow, K.: karyotype, RBC: Red blood cell, TD:transfusion dependency, LEN: lenalidomide, ESA: erythropoiesis stimulating agent, HMA: hypomethylating agents, m: month

The cytogenetic analysis confirmed an isolated del 5q in a majority of patients (61%). Twenty-four percents of patients harbored del 5q and an additional chromosomal abnormality and 11% of patients had a complex karyotype including del 5q. An additional 15 patients (4%) had del 5q only documented by FISH. Data on *TP53* mutations were limited and only available in 10% of the patients, 7 patients harboring a *TP53* mutation. Ninety percent of the patients were RBC transfusion dependent at the initiation of LEN. Use of prior treatments before LEN was uncommon with 39% of patients exposed to erythropoiesis stimulating agents (ESAs) and only 3% previously treated with HMAs, reflecting the differences of indication and access to the drugs in the different countries.

54% of patients responded to LEN with a median durations of treatment of 9.5 months overall, and 17 months for responding patients. Responding patients tended to have non-complex cytogenetics (response rate 57% vs 24%, p<0.001) and experienced a more prolonged survival after LEN initiation (median OS 50 months vs 28 months, p<0.001). LEN treatment schedules varied but most patients were treated using either the 10mg/d for 3 weeks followed by 1 week rest (76% of the patients), or the 5mg/d continuous schedule (23% of the patients), as previously described in the related publications[5, 12]. Thus, we decided to group patients by comparing the initial daily dose of the drug (i.e. 10mg vs less than 10mg). Of note, 8 patients had combinations of LEN and ESA. Data on cytogenetic response were available for 207 patients with 83 pts achieving a cytogenetic CR or PR (40%).

At the time of LEN failure, 141 patients stopped LEN for lack of efficacy (36%, median duration of LEN 5 months), 152 pts for loss of HI without bone marrow progression (39%, median duration of LEN 17 months), 56 pts stopped LEN due to toxicities (14%, median duration of LEN 4 months), and 43 patients (11%) progressed to AML or RAEB-2 (including 23 responders, median duration of LEN 7 months). The supplemental figure 1 shows the patient cohorting diagram for the study. After stopping LEN, an additional 109 patients progressed to AML during follow-up with a 2-year probability of progression of 34% (supplemental figure 2).

### Outcome of patients after failure of Lenalidomide

**Figure 1:**
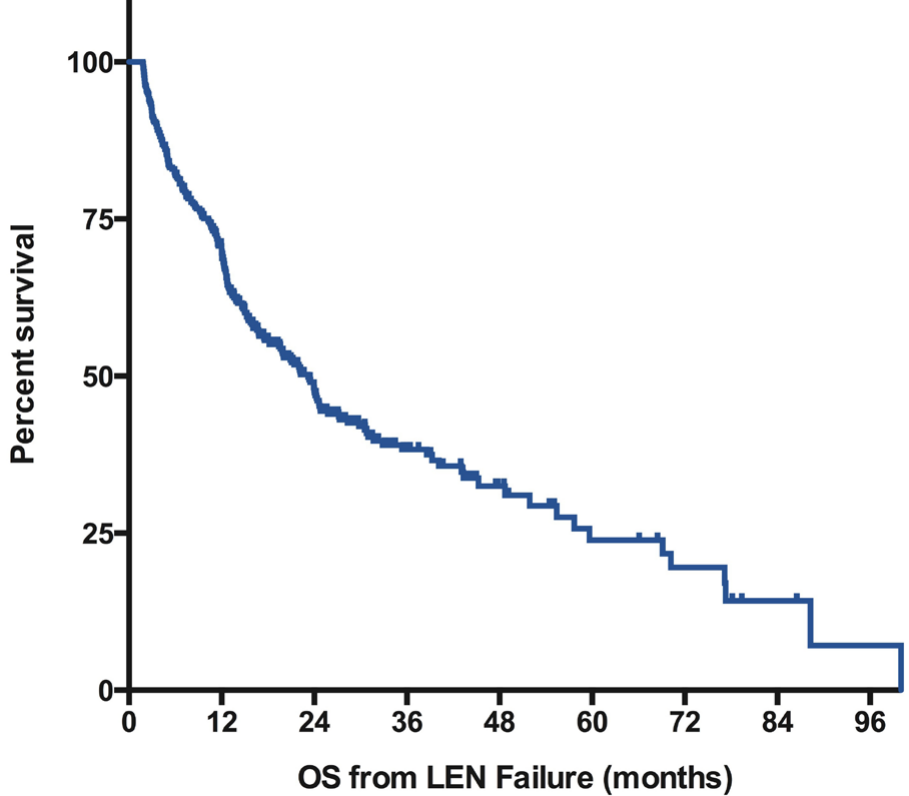
Overall Survival after failure of lenalidomide Survival is defined from documentation of failure to death of any cause or last-follow-up and is expressed in months. , LEN: lenalidomide.

**Figure 2:**
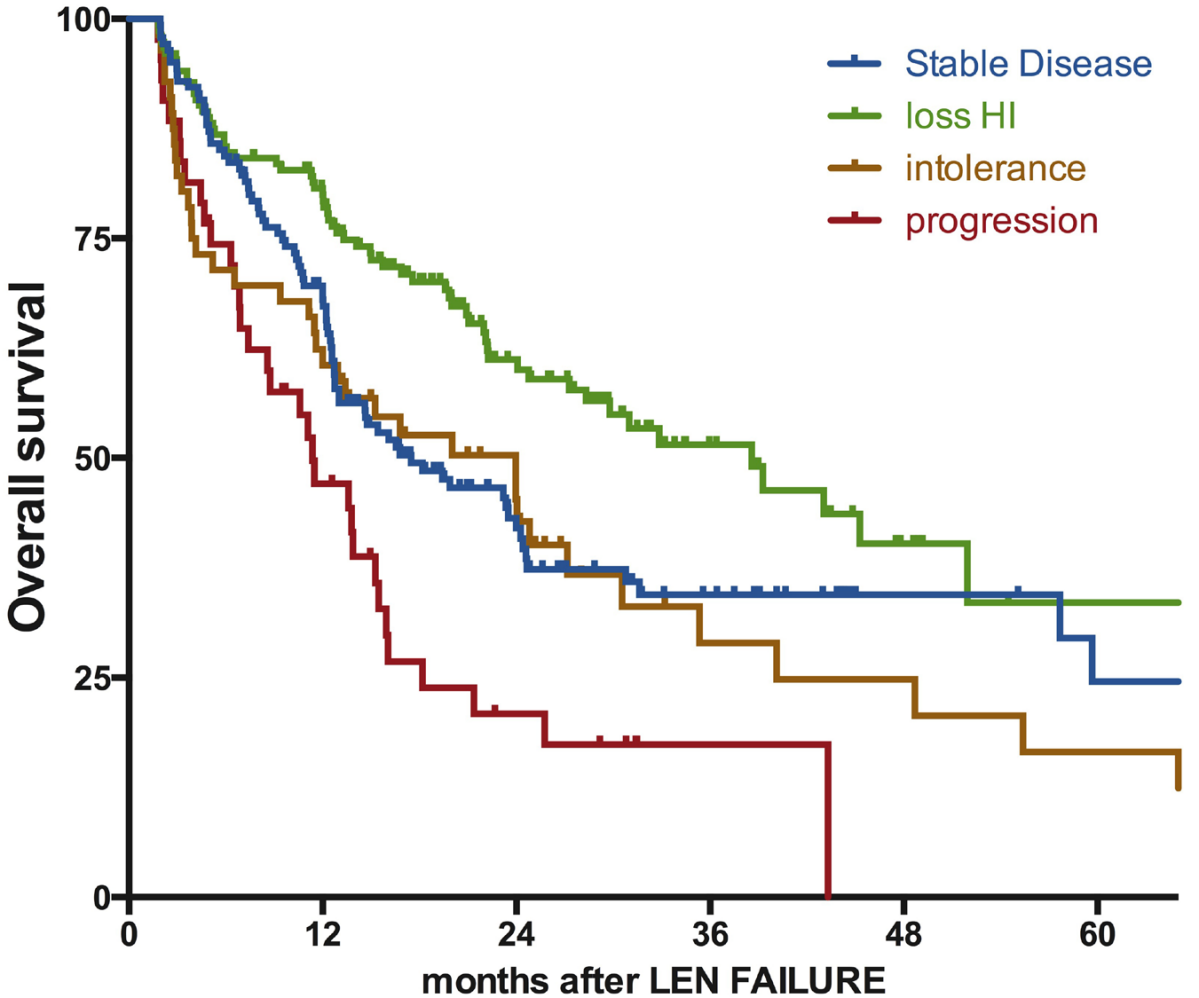
Impact of the type of failure on the outcome after failure of lenalidomide Survival is defined from documentation of failure to death of any cause or last-follow-up and is expressed in months. SD: stable disease, loss of HI: loss of hematologic improvement without bone marrow progression, PD: progressive disease at failure (to RAEB-2 or AML), LEN: lenalidomide.

At last follow-up, 170 patients were alive and 222 had died. With a median follow-up of 22 months for survivors (range: [5-87]), the median overall survival was 23 months (IC95% [20-27]) with a 5-year probability of survival of 24% (figure 1). Patients with isolated del 5q (n=230) had a median survival of 24 months (95%CI [15-33]) and patients with a single additional chromosomal aberration (n=105) had a median OS of 22 months (95%Ci [16-28]). In contrast, patients with complex karyotypes including a del 5q (n=42) had a shorter survival with a median OS of 15 months (95%Ci [9-22]). Considering the type of LEN failure, patients with relapse/secondary loss of HI (n=152) had the best outcome with a median OS of 39 months after LEN failure (95%CI [27-51]) followed by patients with LEN intolerance (n=56, median OS 23 months 95%CI [14-34]), and patients without response (n=141, median OS 17m 95%CI [11-24]). As expected, patients with progression to RAEB-2 or AML had the worst prognosis (n=43, median OS 11m 95%CI [6-17]) (figure 2). Of note, in the subgroup of patients with non-adverse cytogenetics and experiencing secondary loss of HI, the median OS was 43 months (95% CI [28-58]).

In univariate analysis, we observed a negative impact on survival of several factors: age > 74 years (median OS 18 months vs. 27 months, p=0.002), history of therapy-related MDS (median OS 18 months vs. 24 months, p=0.08), presence of excess of blasts at the time of initiation of LEN (median OS 15 months vs. 24 months, p=0.02), presence of complex cytogenetics (median OS 15 months vs. 24 months, p=0.012), absence of response to LEN (median OS 17 months vs. 27 months, p=0.04), reason for LEN failure (progression vs other; median OS 11 months vs. 24 months, p<0.001 ). We did not observe any significant impact of gender, region (US vs. Europe), clinical trial participation, cytogenetics (isolated del 5q vs. 1 additional aberration), RBC transfusion dependence, IPSS (low vs. Intermediate-1), LEN schedule, or LEN duration. Our findings were confirmed in a subgroup analysis performed for patients treated with LEN for at least 6 months (median OS from failure of 39 months for loss of HI vs. 17 months for SD, 12 months for intolerant patients and 14 months for progression, p<0.001, supplemental figure 3). We performed a subgroup analysis for patients with available cytogenetic response data (n=207). Achieving a prior cytogenetic CR or PR had no impact on outcome after failure of LEN (median OS of 17 months vs. 15 months for patients without cytogenetic response, p=NS). In the small subgroup of patients with TP53 mutations, we did not observe any significant difference of survival with 1-year probability of OS of 57 and 59% respectively. Of note, 5 of the 7 mutated patients were allotransplanted in salvage.

The multivariate model included all the above mentioned variables showing an impact on outcome (table 2). We confirmed the detrimental effect of age (HR 1.64, 95%CI [1.24 - 2.18], p=0.001), bone marrow blast excess at LEN initiation (HR 1.41, 95%CI [1.03 – 1.93], p=0.03), bone marrow progression at LEN failure (HR 1.9, 95%CI [1.14 – 3.23], p=0.01), and Complex karyotype (HR 1.65, 95% CI [1.07-2.55], p=0.03). We also performed a separated model to analyze the cumulative incidence of AML in patients without progression at the time of LEN failure. As shown in supplemental table 2, complex karyotype, bone marrow blast excess at LEN initiation, and treatment with HMA prior to LEN initiation had a detrimental impact on the incidence of AML. There was no documented impact of age, use of ESA prior to LEN, response to LEN. The duration of exposure to LEN (+/- 6 months) had a significant impact in univariate analysis that was not confirmed in the multivariate model.

**Table 2:** Multivariate analysis of outcome after lenalidomide failure.

Variable	Median OS	HR	95%CI	*p* value
Age below 75y Age 75+	27m 18m	1 1.64	[1.24 - 2.18]	**0.001**
Adverse K no Adverse K yes	24m 15m	1 1.65	[1.07-2.55]	**0.03**
RAEB no RAEB yes	24m 18m	1 1.41	[1.03 – 1.93]	**0.03**
SD Loss HI Intolerance PD at failure	17m 39m 24m 11m	1 .64 1.09 2.367	[0.36–1.15] [0.68–1.77] [1.609–3.481]	.14 .73 **0.01**
No response to LEN Response to LEN	17m 27m	1 1.04	[0.64-1.70]	0.86

OS: overall survival, HR: Hazard ratio, CI: confidence interval, y: year, m: month, adverse K: adverse karyotype (per IPSS classification), RAEB: refractory anemia with excess of blasts (here limited to 5% to 10% bone marrow blasts), SD: stable disease, loss of HI: loss of hematologic improvement without bone marrow progression, PD: progressive disease at failure (to RAEB-2 or AML), LEN: lenalidomide.

### Impact of treatment strategies after Lenalidomide failure

**Figure 3:**
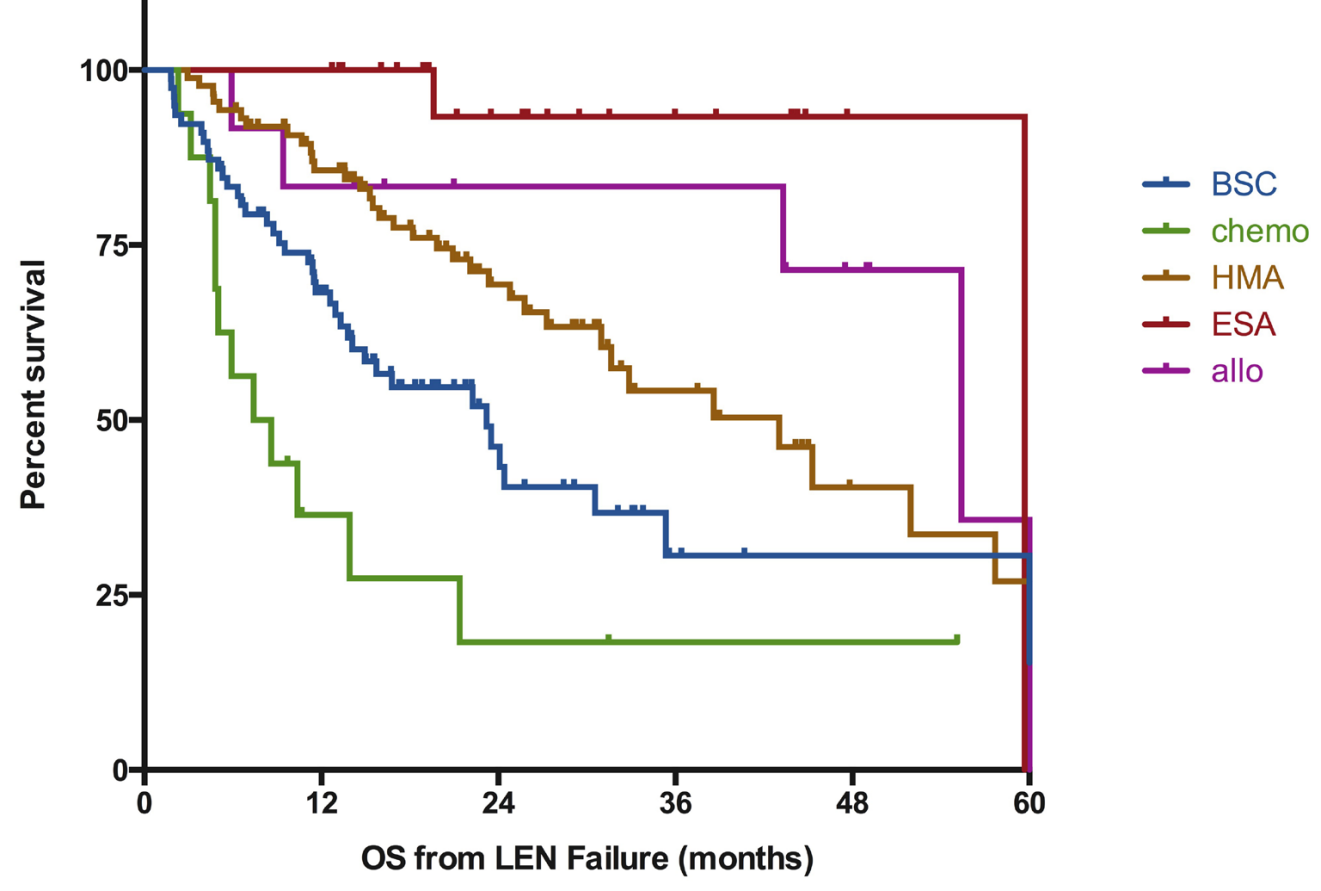
Impact of conventional therapies on outcome after failure of lenalidomide Survival is defined from documentation of failure to death of any cause or last-follow-up and is expressed in months. BSC; best supportive care, chemo: chemotherapy (including AML like induction regimen or lower dose standard chemo), HMA: hypomethylating agents, ESA: erythropoiesis stimulating agents, Allo: allogeneic transplantation, LEN: lenalidomide.

Details of the first treatment given after LEN failure were available in 232 patients (59% of the cohort, figure 3). Patient’s characteristics for each treatment group are described in supplemental table 1. Best supportive care was the only treatment given for 78 patients (34% of the patients with available data) with a median survival of 23 months (95%CI [18-29] months). Patients receiving any active treatment had prolonged survival compared to those receiving BSC only (median OS 49 months vs. 23 months, p=0.003) but the impact of treatment varied with treatment type. Consistent with the dismal outcome of patients with disease progression, patients treated with chemotherapy (n=16) had the shortest overall survival (7 months) with 3 of the 12 patients with available data achieving CR (25%). The larger group of patient (n=91, 39%) received HMA, with 71% of them receiving azacitidine and 29% receiving decitabine. Their overall survival was improved as compared to BSC in univariate analysis (median OS 39 months vs. 23 months, p=0.004) and in a multivariate model (Supplementary Table 3, HR=0.34 95%CI [0.21-0.56]), p=0.002). Of the 71 pts treated with HMA with evaluable response, 32 (45%) achieved a clinical response including 11 CR or PR (15%). The median OS of the 18 patients who received HMA in the context of progressive disease was 16 months (p=0.13 compared to intensive chemo). Interestingly, 4 out of 5 patients with TP53 mutation responded to HMA therapy and 3 bridged to allogeneic transplantation. A group of 22 patients was treated with ESAs, including 10 who already had received ESAs prior to LEN. The median overall survival of the ESAs group was 59 months, with no notable difference of survival between patients with or without prior exposure to ESAs. Of note, only 2 patients responded to ESAs therapy after LEN (2/18 with available data) including 1 patient with prior exposure to ESAs before LEN. Allogeneic transplantation was performed in 30 patients with transplantation performed immediately after LEN failure in 12 cases and after other salvage therapies in the remaining 18 cases. Median OS was 52 months (55 months for patients allotransplanted directly after LEN failure) and was not significantly improved as compared to patients treated with other strategies (median OS 35 months, p=0.7). As seen on figure 3, we did not observe any long term survival plateau for allotransplanted patients. Finally, 13 patients (6%) were treated with other modalities (Thalidomide (n=4), Antithymocyte globulin (n=3), danazol (n=1), ruxolitinib (n=1), clinical trials (n=4)) and those numbers were too low to evaluate outcome.

## DISCUSSION

This is the first large study focused on the outcome of MDS patients with deletion 5q after LEN failure. We report a median survival of 23 months for patients treated with best supportive care. This will help to define a baseline of future investigations. We showed that outcome was heterogeneous and influenced by patients’ characteristics and type of LEN failure. Finally, we observed that standard of care strategies used after LEN may have some potential benefit in patients treated with HMAs, acknowledging the limitation of a non-prospective design.

As previously mentioned, the combination of del 5q and LEN treatment is a unique paradigm in the MDS field. We know from prior studies of patients with del 5q that conventional treatments, ESAs for instance, are probably less effective in these patients as compared to those without the deletion [20] and that LEN is able to trigger deeper responses with cytogenetic remission, potentially modifying the natural history of the disease. This may explain why only a minority of our patients were exposed to ESAs prior to LEN, as a significant number of clinicians will choose to directly treat with LEN. This point is still a matter of debate in the hematology community. In our hands and keeping in mind the potential limitations of our cohort, we observed a similar overall survival calculated from diagnosis for patients previously treated with ESAs or not (median OS 82 months vs. 77 months respectively, p=0.16).

We need to acknowledge that the retrospective nature of our study exposes to some limitations and bias but, indeed, there are few other ways to evaluate standard of care strategies in these settings. In our study, the large number of patients and the high proportion of patients included in clinical trials and prospective compassionate programs limits the risk selection bias. Moreover, patients from the centers databases were consecutively registered and cases included in our study were only selected to fulfill the inclusion criteria listed in the methods section. Despite the high number of patients, some potentially interesting variables were not available in a majority of cases and could not integrated in the analyses: comorbidities scorings were only documented for 95 patients and *TP53* mutation status was only available for 39 patients. The impact of *TP53* mutations[21, 22] and P53 expression[23] in the progression of MDS seems especially important in the context of 5q deletion. The frequency of *TP53* mutation in our small subgroup (7/39 patients harboring a mutation, 18%) matches what has been presented in the prior studies dedicated to del 5q MDS [21, 22] as well as what was described in large-scale genomic studies [24]. This point is interesting, as we may have expected a slightly more elevated incidence in a group of patients selected for LEN failure. The relatively high response to salvage HMA is in line with the recent New England Journal of Medicine publication[25].

Our multivariate analysis model for overall survival showed a detrimental impact of variables expected to be associated with a negative outcome: older age, cytogenetic complexity, and presence of an excess of blasts at the initiation of lenalidomide. Interestingly, we also showed that treatment related variables also have some influence. The progression to a more aggressive presentation (RAEB-2 or AML) strongly impaired the chances of long-term survival and was associated with a poor outcome when treated with conventional therapies (7 months for conventional chemo, 16 months for hypomethylating agents). In contrast, patients who relapsed after achieved an erythroid response seemed to have a more favorable outcome with a median OS of 39 months. Even if it can be argued that it represents a selection bias, we can speculate that it may reflect the changes in the natural history of the disease induced by LEN. This is also suggested by the absence of impact on outcome of the duration of LEN by itself.

The analysis of the treatment strategies used after LEN failure must be interpreted with caution as the choice of therapy was driven by the type of failure and by the patient medical condition (Supplementary Table 1). Patients with progression to AML were treated aggressively and had a dismal outcome, while younger patients with less comorbidities and stable lower-risk disease were more commonly treated with allogeneic transplantation and experienced a relatively prolonged survival. Of note, we were not able to demonstrate a significant survival benefit of allogeneic transplantation in either the group of patients allotransplanted upfront or in the whole group of allotransplanted patients, acknowledging that one of the potential limitation of our analysis remains the relatively small number of patients. Finally, we showed a relatively favorable outcome of patients treated with HMAs. It is important to notice that the majority of patients exposed to HMA did not had bone marrow progression. Median overall survival was strongly influenced by progression in this subgroup: 45 months for patients without progression as compared to 16 months for patients treated with HMA for progression (p<0.001). Moreover, we did not observe any long-term survival plateau (as shown in figure 3). This improved outcome for the sequence LEN / HMA has already been suggested in non-del5q MDS patients [26] and should warrant further investigation with HMA used as a potential backbone of combinations therapies. The type and schedule of HMA that could be used remain an open question.

In conclusion, our study defined the expected baseline for future clinical investigation in the settings of MDS with 5q deletion experiencing LEN failure. Despite the very good results of LEN in this group of patient, not all patients respond and responses are transient. The survival of this group of usually younger patients is relatively short for patients that only had access to supportive care. Hypomethylating agents appeared here as a potential backbone of future investigations. Finally, our results stress again the need to focus basic and translational research on new ways to eradicate the 5q clone.

## PATIENTS AND METHODS

### Patient selection

This study was an international collaboration which included patient treated in clinical trials [5, 6, 12], prospective compassionate use programs [27], as well as patients followed in MDS registries in the different centers. Patients were eligible for the study if they fulfilled the following criteria: 1: diagnosis of MDS according to WHO 2008 classification [28] 2: presence of a del 5q confirmed by conventional cytogenetics and/or fluorescent in situ hybridization (FISH) techniques 3: treatment with single agent LEN for the MDS and 4: documentation of LEN failure (see below for the definition of LEN failure). All patients gave consent for the use of their clinical and biological data and Yale University internal review board has approved the study.

Patients with higher risk disease refractory anemia with excess of blast-2 (RAEB-2) or acute myeloid leukemia (AML) were excluded as were patients treated with combinations of LEN with other active treatments (chemotherapy, HMA). However, combinations with hematopoietic growth factors or iron chelation therapies were accepted. Patients treated with LEN as remission maintenance therapy, for instance after allogeneic transplantation, were also excluded from the analysis.

Cytogenetic risk was assessed based on International Prognostic Scoring System (IPSS) [29].

### Definition of Lenalidomide failure

Clinical and cytogenetic responses were evaluated according to the international working group 2006 MDS criteria [30]. The initial intent was to treat patients for 3 to 6 months with disease assessment every 8 weeks. Responding patients were treated until documentation of treatment failure. We defined 4 different categories: absence of response, bone marrow progression during treatment with or without prior response, secondary failure (loss of a prior hematological response without bone marrow progression), and intolerance (treatment stopped related to adverse event, with or without prior response).

### Statistical methods

Data were summarized by frequency and percentage for categorical variables. For continuous variables, the median and range were computed. All results are presented with their 95% confidence intervals. Statistical tests were two-sided at the 5% level of significance. To investigate the association between continuous variables and categorical variables, univariate statistical analyses were performed using non-parametric Wilcoxon rank sum test, Chi square test or Fisher’s exact test when appropriate. Survival rates were estimated by the Kaplan-Meier method and log-rank test. Overall survival (OS) was measured from the date of LEN failure until death from any cause with observation ending at the date of last contact for patient last known to be alive. Patients without events were censored at the date of last follow up. Multivariate analyses were performed using a Cox proportional hazards method. All variables with p-value below 0.15 in univariate analysis were included in the Cox model using a stepwise procedure selection. Statistical tests were performed using SPSS 21.0 and graphs were designed using PRISM 6 software.

## SUPPLEMENTARY MATERIALS FIGURES AND TABLES


