# Supplementary material for: Outcome of patients treated for myelodysplastic syndromes with 5q deletion after failure of lenalidomide therapy
Source: Oncotarget. 2017 Jun 14;8(47):81926–35. doi: 10.18632/oncotarget.18477 (PMC5669859; doi:10.18632/oncotarget.18477)
Supplement: Supplementary file 1 [file oncotarget-08-81926-s001.pdf]

# Outcome of patients treated for myelodysplastic syndromes with 5q deletion after failure of lenalidomide therapy

## SUPPLEMENTARY MATERIALS

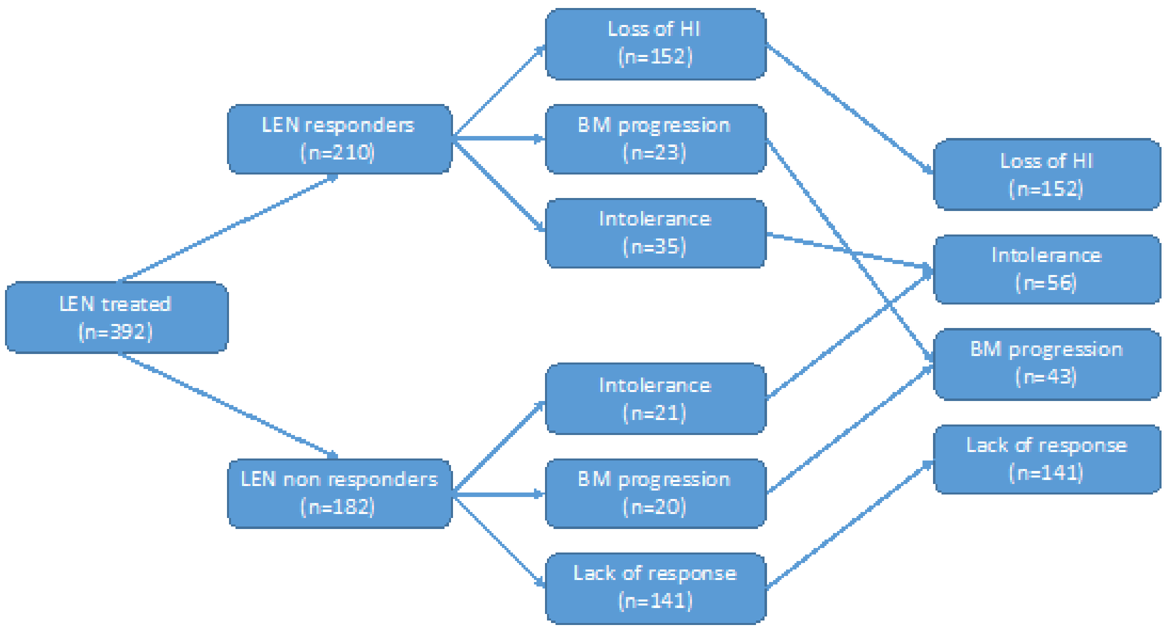


**Supplementary Figure 1: Cohort attribution diagram.** LEN: lenalidomide, BM: bone marrow, HI: hematologic improvement

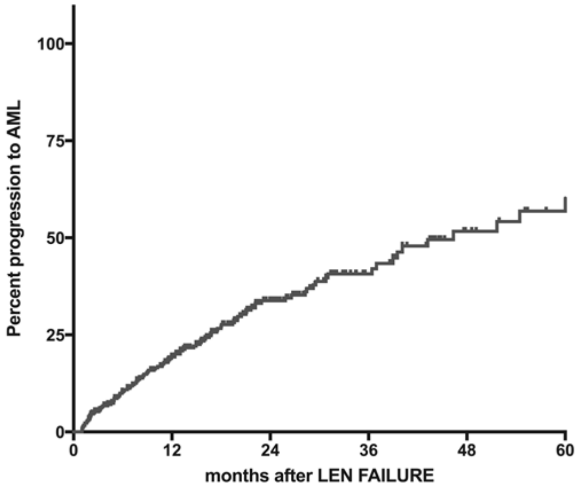


**Supplementary Figure 2: Cumulative Incidence of AML after Lenalidomide Failure.** Time to AML is expressed in months and is defined from the time of documentation of Lenalidomide (LEN) failure to time of documentation of progression. Patients without progression were censored at last follow-up. Patients with progression to AML at the time of documentation of LEN failure were excluded from the analysis.

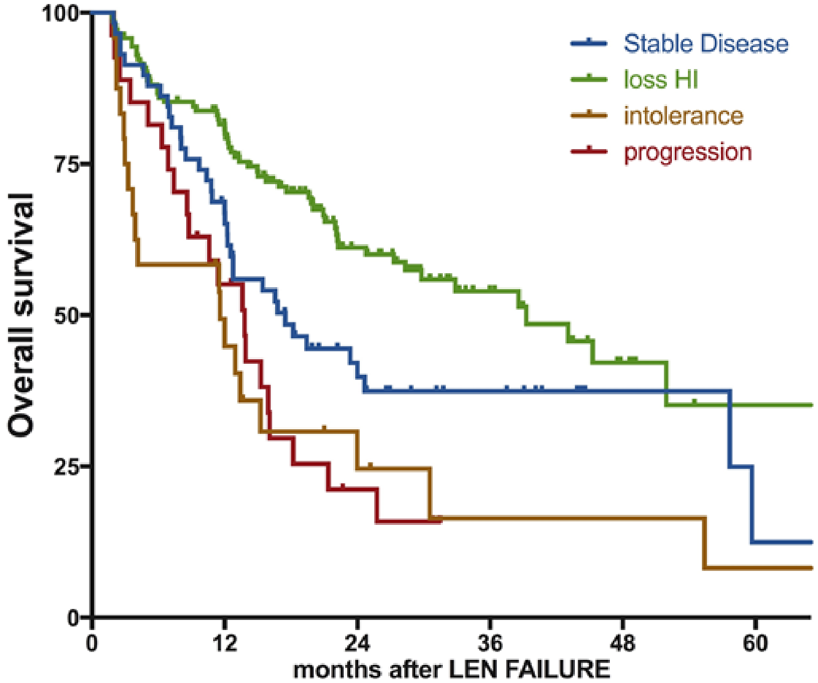


**Supplementary Figure 3: Impact of the type of failure on the outcome after failure of lenalidomide: subgroup analysis in patients treated with 6 months or more of lenalidomide Survival analysis performed on the subgroup of patients treated for at least 6 months with lenalidomide before failure.** Survival is defined from documentation of failure to death of any cause or last-follow-up and is expressed in months. SD: stable disease, loss of HI: loss of hematologic improvement without bone marrow progression, PD: progressive disease at failure (to RAEB-2 or AML), LEN: lenalidomide. median OS from failure of 39 months for loss of HI vs. 17 months for SD, 12 months for intolerant patients and 14 months for progression, ($p<0.001$).

**Supplementary Table 1: Patients characteristics in each treatment groups.**
BSC; best supportive care, chemo: chemotherapy (including AML like induction regimen or lower dose standard chemo), HMA: hypomethylating agents, ESA: erythropoiesis stimulating agents, Allo: allogeneic transplantation, LEN: lenalidomide.In the allogeneic transplantation group, patients characteristics refers to patients allotransplanted upfront and an additional 18 pts were allotransplanted after other treatments. *: indicated a statistically significant difference as compared to BSC ($p<0.05$)

| | BSC | HMAs | ESAs | Chemo | Allo upfront |
|---|---|---|---|---|---|
| **N=** | 78 | 91 | 22 | 16 | 12 |
| **Median age** | 74 (45-90) | 67 (38-85)* | 70 (50-81) | 66 (55-83)* | 59 (46-69)* |
| **Male Gender M/F** | 20 (26%) | 16 (18%) | 6 (27%) | 10 (69%) | 4 (33%) |
| **WHO classification** | | | | | |
| RAEB-1 | 3 (4%) | 30 (33%)* | 6 (27%)* | 5 (31%)* | 1 (8%) |
| **Therapy related MDS** | 7 (9%) | 16 (18%) | 5 (22%) | 3 (19%) | 1 (8%) |
| **Median BM blast count (%)** | 2 (0-9) | 3 (0-9) | 3 (0-9) | 4 (0-9) | 2 (0-5) |
| **Deletion (5q)** | | | | | |
| isolated | 59 (76%) | 53 (58%) | 19 (86%) | 6 (38%) | 8 (67%) |
| Del(5q)+1 aberration | 13 (17%) | 18 (20%) | 3 (14%) | 4 (24%) | 2 (17%) |
| Complex K including del(5q) | 6 (7%) | 20 (22%)* | 0* | 6 (38%)* | 2 (17%) |
| **RBC TD before LEN** | 63 (81%) | 79 (87%) | 17 (77%) | 13 (81%) | 9 (75%) |
| **Use of ESA before LEN** | 35 (45%) | 20 (22%)* | 10 (45%) | 5 (31%) | 4 (33%) |
| **Use of HMA before LEN** | 3 (4%) | 3 (3%) | 1 (5%) | 5 (31%) | 0 |
| **LEN response** | 48 (62%) | 53 (58%) | 12 (55%) | 6 (38%)* | 10 (83%) |
| **LEN initial dose 10mg daily** | 51 (65%) | 75 (82%) | 11 (50%) | 11 (69%) | 9 (75%) |
| **LEN duration** | 9 (1-68) | 10 (1-64) | 9 (1-55) | 7 (0-28) | 16 (0-65) |
| **Progression at LEN failure** | 11 (14%) | 18 (20%)* | 0* | 10 (63%)* | 1 (8%) |

**Supplementary Table 2**: **Multivariate analysis model of overall survival after lenalidomide failure: Comparison of BSC and HMA populations.**

Subgroup analysis focused on patients treated with best supportive care and hypomethylating agents (HMA). Cox model with Bonferroni correction for multitesting has been applied. All variables with an impact in univariate analyses were integrated in the model.

| Variable | HR | 95%CI | p value |
|---|---|---|---|
| Age below 75y<br>Age 75+ | 1<br>1.8 | [1.24 - 2.18] | 0.056 |
| Adverse K no<br>Adverse K yes | 1<br>3.6 | [1.07-2.55] | **0.004** |
| RAEB no<br>RAEB yes | 1<br>1.12 | [0.51 – 1.61] | 0.72 |
| No PD at failure<br>PD at failure | 1<br>5.96 | [3.38–10.6] | **<0.01** |
| BSC<br>HMA | 1<br>0.34 | [0.21-0.56] | 0.002 |

**Supplementary Table 3**: **Cox model analyzing the cumulative incidence of AML in 5q patients experiencing LEN failure.**
Patients experiencing disease progression at the documentation of progression were excluded from the analysis. Survival is expressed in months. CI AML: cumulative incidence of AML

| Variable | Median CI AML | HR | 95%CI | p value |
|---|---|---|---|---|
| No prior HMA<br>Prior HMA | 62m<br>26m | 1<br>3.65 | [1.51 – 8.77] | **0.004** |
| Adverse K no<br>Adverse K yes | 66m<br>44m | 1<br>2.35 | [1.25-4.42] | **0.008** |
| RAEB no<br>RAEB yes | 71m<br>57m | 1<br>1.99 | [1.27 – 3.13] | **0.03** |
| Duration of LEN<6m<br>Duration of LEN 6m+ | 54m<br>69m | 1<br>0.72 | [0.47-1.10] | 0.13 |
